# Correlation analysis of Hashimoto’s thyroiditis with papillary thyroid carcinoma occurrence and its central lymph node metastasis: a single center experience

**DOI:** 10.3389/fendo.2024.1420998

**Published:** 2025-02-06

**Authors:** Kang Sun, Xiaoming Wang, Dexuan Chen, Chaoqun Ma

**Affiliations:** ^1^ Department of General Surgery, Jiangsu Province Hospital of Chinese Medicine, Affiliated Hospital of Nanjing University of Chinese Medicine, Nanjing, China; ^2^ Department of General Surgery, The First Affiliated Hospital of Anhui University of Chinese Medicine, Hefei, China

**Keywords:** papillary thyroid carcinoma, Hashimoto’s thyroiditis, central lymph node metastasis, multivariate analysis, risk factors

## Abstract

**Purpose:**

This study investigates the clinicopathological characteristics of papillary thyroid carcinoma (PTC) with coexisting Hashimoto’s thyroiditis (HT) and further explores the risk factors for central lymph node metastasis (CLNM) in PTC.

**Method:**

A retrospective analysis was conducted on 415 PTC patients who underwent surgical treatment for thyroid cancer at the First Affiliated Hospital of Anhui University of Chinese Medicine from 2016 to 2022. Clinicopathological features were compared between PTC patients with and without HT. Univariate and multivariate logistic regression were used to analyze the risk factors of CLNM.

**Result:**

The PTC+HT group had a higher proportion of female patients (85.5%) than the PTC group (P<0.05). Univariate analysis revealed no statistically significant difference between the two groups in eight aspects (all P>0.05). Multivariate analysis showed that HT was positively associated with the total number of central lymph node (CLN) dissected, Thyroid-stimulating hormone (TSH), Thyroid peroxidase antibody (TPOAb), and Thyroglobulin antibodies (TgAb), while identified as a protective factor against invasion with an odds ratio of 0.422 (95%CI 0.209-0.853, P=0.016). Through univariate and multivariate logistic regression, we proved that tumor position, Capsule + Extrathyroidal extension (ETE), multifocal tumors, and the total number of CLN dissected were independent risk factors for CLNM. Multiple linear regression analysis told us that invasion (β= 0.093, p=0.048) had a positively predictive impact on CLN positive rate.

**Conclusion:**

Female PTC patients are more prone to concurrent HT, which elevates the levels of TSH, TPOAb, and TgAb. HT not only promotes the longitudinal growth of nodules and PTC development, but also reduces the risk of invasion and CLNM. Therefore, we posit that the impact of HT on PTC patients is a “double-edged sword”. Isthmus, Capsule + ETE, multifocality, age < 55 years old, and male are high-risk factors for CLNM in PTC, while HT is regarded as a protective factor. Capsule + ETE is the primary risk factor affecting the CLN positive rate.

## Introduction

Thyroid carcinoma is the predominant malignant disease affecting the endocrine system, characterized by a favorable prognosis and high overall survival rate, particularly in cases of papillary thyroid carcinoma (PTC) (1). However, with the continuous improvement of diagnostic tools ——such as fine needle aspiration, high-resolution ultrasound, thyroid-specific antibody testing, and genetic testing——the morbidity of thyroid cancer has increased dramatically in recent years (2). Hashimoto’s thyroiditis (HT), also known as chronic lymphocytic thyroiditis or autoimmune thyroiditis, is the most prevalent autoimmune thyroid disease and the primary cause of hypothyroidism in iodine-sufficient regions worldwide (3). Studies show that approximately one-third of individuals diagnosed with PTC also have HT, and this prevalence is increasing rapidly (4–6).

Since Rudolf Virchow initially posited the link between chronic inflammation and cancer development in 1863, a growing number of studies have substantiated that chronic inflammation can contribute to cancer progression, such as inflammatory bowel disease with colon cancer, chronic viral hepatitis with liver cancer, as well as chronic gastritis induced by Helicobacter pylori infection with gastric cancer (7–13). The association between HT and PTC was first described by Dailey et al. (14) in 1955. Subsequently, much research has been conducted on this topic, but a consensus has yet to be reached. So, does the presence of HT increase the risk of developing PTC? And does HT contribute to the occurrence of lymph node metastasis in PTC? Many studies have confirmed that HT can promote the occurrence of PTC, and male, age ≤45 years, and tumor diameter > 1cm are risk factors for CLNM in PTC patients (5, 15, 16). However, there is some disagreement about whether HT increases the risk of CLNM in PTC patients (17, 18). Therefore, based on the analysis of the clinical characteristics of PTC patients with HT, this study will further focus on the impact of HT on the risk rate of CLNM in PTC patients. We performed a retrospective analysis of clinical and pathological data from 415 patients who underwent surgery for PTC, with a focus on the clinical characteristics of PTC in combination with HT.

## Materials and methods

### Patients

This study comprehensively evaluated all patients who underwent thyroidectomy for PTC at the Second Department of General Surgery, First Affiliated Hospital of Anhui University of Chinese Medicine, from January 2016 to December 2022.

Inclusion criteria: (1) First-time surgical intervention for thyroid cancer; (2) Postoperative pathological diagnosis confirming PTC; (3) No prior occurrence of thyroid or cervical lymph node disorders; (4) No history of other malignant tumors; (5) Availability of complete clinical and pathological data.

Exclusion criteria: (1) Metastatic thyroid cancer; (2) Postoperative pathology showing other types of thyroid cancer, such as follicular or medullary carcinoma; (3) Previously diagnosed as thyroid or cervical lymph node diseases; (4) History of other malignant tumors or current coexistence of other malignancies; (5) Incomplete clinical or pathological data.

After screening, a total of 415 cases were selected for inclusion in this study. Based on postoperative pathological examinations, the patients were categorized into two groups: the PTC with HT group (PTC+HT) and the PTC without HT group (PTC). The ethical review for this project was approved and registered by the Medical Ethics Committee of the First Affiliated Hospital of Anhui University of Chinese Medicine.

### Preoperative examination

The blood samples from all patients were consistently analyzed at the Test Center of the First Affiliated Hospital of Anhui University of Chinese Medicine. The testing parameters included serum Thyroid-stimulating hormone (TSH), serum Free triiodothyronine (FT3), serum Free thyroxine (FT4), Thyroglobulin antibodies (TgAb), and Thyroid peroxidase antibody (TPOAb). All preoperative thyroid color ultrasound examinations were conducted by physicians at the Ultrasound Center of the First Affiliated Hospital of Anhui University of Chinese Medicine.

### Surgical methods

All patients underwent either unilateral or bilateral thyroidectomy, accompanied by isthmus and ipsilateral central lymph node dissection. In cases where biopsy cytology or intraoperative frozen section revealed lateral lymph node metastasis, functional lateral lymph node dissection was performed simultaneously. All surgeries were executed in an open situation by highly qualified surgeons in our department. No serious postoperative complications were observed in any of the patients after surgery. The cervical lymph node zoning and extent of lymph node dissection in each region strictly adhered to the guidelines established by the American Thyroid Association (19).

### Pathological diagnosis

The pathological examination results of all patients were uniformly reported by the pathology department of our hospital. Each pathology report offered a detailed description of the tumor, covering its position, maximum diameter, invasion of the thyroid capsule, number of lesions, lymph node zoning and counts, as well as lymph node metastasis. The pathological staging followed the 2017 8th edition of the American Joint Committee on Cancer (AJCC) Staging Guidelines for Differentiated Thyroid Cancer (20). All patients were diagnosed with PTC in their pathological reports.

### Diagnostic criteria and grouping

The evaluation of thyroid capsule invasion was not solely reliant on subjective observation. Rather, it was a synthesis of the surgeon’s notes in the operative records and the final pathology reports. In instances where there was a discrepancy, the pathological results were prioritized. The diagnostic criteria for HT were as follows: postoperative pathology showed that thyroid tissue appeared enlarged, grayish, and firm, thyroid cells in some patients displayed enlarged and thickened, leading to distinctive Hürthle cells. Additionally, the stroma was heavily infiltrated by hematopoietic monocytes, predominantly lymphocytes alongside some plasma cells, while lymphoid follicles and active germinal centers were formed. Patients whose pathological reports matched the above description were categorized into the PTC+HT group. The remaining patients were placed in the PTC group.

### Observed index

Retrospective data analysis was used to compare the clinicopathological characteristics between the two groups. The clinicopathologic features mainly encompassed age, gender, position, maximum tumor diameter, presence of microcarcinoma, presence of calcification, anteroposterior to transverse diameter ratio (A/T), invasion, number of lesions, number of central lymph node (CLN) dissected, number of central lymph node metastasis (CLNM), pathological stage, preoperative thyroid function indicators (including FT3, FT4, TT3, TT4, TSH, TPOAb, TgAb), etc. Both capsular invasion and extrathyroidal extension (ETE) indicated invasion.

### Statistical analysis

The data were processed using SPSS statistical software and R language software. Measurement data were presented as mean and standard deviation. For normally distributed data, t-test and ANOVA were employed for comparative analysis. Categorical data were represented as percentages (%), compared by χ^2^ test and rank-sum test. Non-parametric tests were used for ordinal and non-normally distributed data. Multivariate linear regression was applied to assess continuous variables, while logistic regression was utilized for binary dependent variables. The predictive value of those factors was measured through the area under the receiver operating characteristic (ROC) curve. P<0.05 was deemed statistically significant in all tests.

## Results

### Basic clinicopathological features of HT group and PTC+HT group

A cohort of 415 patients diagnosed with PTC was selected in this study, comprising 96 males and 319 females, resulting in a male-to-female ratio of approximately 1:3. The age distribution of the patients ranged from 15 to 78 years, with a mean age of 45.23 ± 12.090 years and a median age of 46 years. Among these patients, 319 cases were younger than 55 years, whereas 96 cases were 55 years or older.

According to the presence or absence of HT, the subjects were divided into two groups. The PTC group comprised 298 patients, including 79 males (26.5%) and 219 females (73.5%), with a mean age of 45.70 ± 12.586 years. In contrast, the PTC+HT group included 117 patients, consisting of 17 males (14.5%) and 100 females (85.5%), with a mean age of 44.05 ± 10.681 years. While the mean age in the PTC group was slightly higher than that in the PTC+HT group, the difference did not reach statistical significance(P>0.05). Simultaneously, a higher proportion of patients under 55 years old was observed in the PTC+HT group (82.1%) compared to the PTC group (74.8%), though this disparity was also not statistically significant(P>0.05). However, the percentage of female patients was higher in the PTC+HT group (85.5%) compared to the PTC group (P<0.05). Analysis of the pathological examination results revealed no statistically significant differences between the two groups across eight factors: position, maximum tumor diameter, presence of microcarcinoma, presence of calcification, invasion, number of lesions, number of positive central lymph node, and pathological staging (all P>0.05). Furthermore, upon dividing the maximum tumor diameter into two groups with a 20mm boundary, no statistically significant difference was observed between the two groups (P>0.05). Conversely, the proportion of A/T >1 in the PTC+HT group (59.8%) was higher than that in the PTC group (44.6%), showing a statistically significant difference (P<0.05). The total number of central lymph node dissected in the PTC+HT group (7.62 ± 5.898) was markedly higher than that in the PTC group (P<0.00). Similarly, the PTC+HT group had a greater percentage of central lymph node with a positive rate compared to the PTC group (P=0.007). The laboratory examination results revealed that the mean values of FT3 before operation in the PTC+HT group were slightly lower than that in the PTC group (P<0.05). The preoperative mean values of TSH, TPOAb, and TgAb all exhibited an increase in the PTC+HT group compared to the PTC group (P<0.05), with the latter two indicating a more pronounced rise. Nevertheless, there were no notable differences in the mean values of FT3, TT3, and TT4 between the two groups before operation (P>0.05). (**Table 1**)

**Table 1:** Comparison of clinicopathological characteristic in PTC patients according to with or without HT.

Group	PTC (n=298)	PTC+HT (n=117)	t/U/χ^2^ value	P
Age	45.70 ± 12.586	44.05 ± 10.681	16088	0.221
<55	223 (74.8%)	96 (82.1%)	2.073	0.150
≥55	75 (25.2%)	21 (17.9%)		
Gender				
Male	79 (26.5%)	17 (14.5%)	6.124	0.013
Female	219 (73.5%)	100 (85.5%)		
Position				
Left	119 (39.9%)	42 (10.1%)	4.713	0.180
Right	135 (45.3%)	50 (12.0%)		
Bilateral	41 (13.8%)	25 (6.0%)		
Isthmus	3 (1.0%)	0 (0%)		
Maximum tumor diameter (mm)	10.66 ± 7.888	9.31 ± 5.871	16339	0.318
<20	256 (85.9%)	107 (91.5%)	1.880	0.170
≥20	42 (14.1%)	10 (8.5%)		
Microcarcinoma				
No	113 (37.9%)	35 (29.9%)	2.010	0.156
Yes	185 (62.1%)	82 (70.1%)		
Calcification				
No	132 (44.3%)	54 (46.2%)	0.054	0.816
Yes	166 (55.7%)	63 (53.8%)		
A/T >1				
No	165 (55.4%)	47 (40.2%)	7.170	0.007
Yes	133 (44.6%)	70 (59.8%)		
Invasion				
No	210 (70.5%)	93 (79.5%)	3.024	0.082
Capsule + ETE	88 (29.5%)	24 (20.5%)		
Number of lesions				
Unifocality	222 (74.5%)	76 (65%)	3.320	0.068
Multifocality	76 (25.5%)	41 (35%)		
The total number of CLN dissected	4.27 ± 3.709	7.62 ± 5.898	11581	0.000
Number of positive CLN	1.19 ± 2.072	1.00 ± 2.316	15566.5	0.056
CLN positive rate (%)	24.96 ± 34.983	13.92 ± 27.887	14786	0.007
Staging				
I	247 (82.9%)	104 (88.9%)	3.626	0.297
II	16 (5.4%)	6 (5.1%)		
III	23 (7.7%)	6 (5.1%)		
IV	12 (4.0%)	1 (0.9%)		
FT3 (pmol/L)	4.37 ± 1.03	4.14 ± 0.64	14994	0.027
FT4 (pmol/L)	12.86 ± 2.08	12.36 ± 2.11	15487	0.077
TT3 (nmol/L)	1.46 ± 0.32	4.42 ± 30.65	16525	0.409
TT4 (nmol/L)	94.41 ± 19.40	93.18 ± 29.43	16278	0.293
TSH (mIU/L)	1.98 ± 1.38	3.09 ± 3.98	12760	0.000
TPOAb (IU/ml)	17.55 ± 86.67	180.85 ± 263.00	5314.5	0.000
TgAb (IU/ml)	30.31 ± 124.52	163.93 ± 254.22	4549.5	0.000

### Multivariate analysis of the effect of coexistent HT in PTC patients

All potential variables related with HT were added to the Logistic regression model. Multivariate analysis displayed that HT was positively associated with the total number of CLN dissected (OR=1.186, 95%CI 1.100-1.280, P<0.001), TSH (OR=1.260, 95%CI 1.043-1.521, P=0.016), TPOAb (OR=1.007, 95%CI 1.004-1.010, P<0.001) and TgAb (OR=1.003, 95%CI 1.001-1.004, P<0.001). Nevertheless, HTemerged as a protective factor against invasion, with an odds ratio (OR) of 0.422, (95%CI 0.209-0.853, P=0.016) (**Table 2**). After stratifying adjustments for age and maximum tumor diameter, we obtained the same outcomes as above.

**Table 2:** Multivariate Logistic regression analysis the effect of coexistent HT in PTC patients.

Variables	Subgroup	Unadjusted			Subgroup	Adjusted		
		OR	95% CI	P		OR	95% CI	P
Age		0.990	0.965-1.015	0.426	<55	1		
					≥55	0.669	0.328-1.367	0.270
Gender	Male	1			Male	1		
	Female	1.256	0.572-2.761	0.570	Female	1.251	0.570-2.745	0.577
Position	Left	1		0.842	Left	1		0.837
	Right	1.108	0.594-2.067	0.747	Right	1.125	0.601-2.103	0.713
	Bilateral	0.000	0.000-.	0.999	Bilateral	0.000	0.000-.	0.999
	Isthmus	0.682	0.244-1.911	0.467	Isthmus	0.686	0.244-1.923	0.473
Maximum tumor diameter (mm)		0.985	0.918-1.055	0.660	<20	1	0.174-1.851	0.348
					≥20	0.568		
Microcarcinoma	No	1			No	1		
	Yes	0.896	0.320-2.510	0.834	Yes	0.890	0.419-1.889	0.762
Calcification	No	1			No	1		
	Yes	0.671	0.378-1.189	0.171	Yes	0.648	0.365-1.152	0.139
A/T >1	No	1			No	1		
	Yes	1.658	0.903-3.047	0.103	Yes	1.651	0.900-3.030	0.105
Invasion	No	1			No	1		
	Capsule + ETE	0.422	0.209-0.853	0.016	Capsule + ETE	0.411	0.202-0.834	0.014
Number of lesions	Unifocality	1			Unifocality	1		
	Multifocality	1.501	0.667-3.380	0.326	Multifocality	1.491	0.666-3.337	0.331
The total number of CLN dissected		1.186	1.100-1.280	0.000		1.182	1.094-1.276	0.000
Number of positive CLN		0.869	0.695-1.087	0.218		0.884	0.708-1.104	0.277
CLN positive rate (%)		0.720	0.159-3.264	0.670		0.691	0.153-3.126	0.632
FT3		0.593	0.338-1.040	0.068		0.587	0.336-1.025	0.061
FT4		1.044	0.798-1.367	0.752		1.044	0.799-1.364	0.753
TT3		1.204	0.498-2.912	0.681		1.198	0.523-2.745	0.669
TT4		0.997	0.972-1.023	0.822		0.998	0.973-1.023	0.851
TSH		1.260	1.043-1.521	0.016		1.259	1.043-1.520	0.016
TPOAb		1.007	1.004-1.010	0.000		1.007	1.004-1.010	0.000
TgAb		1.003	1.001-1.004	0.000		1.003	1.001-1.004	0.000

### Univariate analysis predictive risk factors of CLNM in PTC patients

Univariate analysis found that age (P<0.001), gender (P=0.016), position (P=0.023), microcarcinoma (P<0.001), calcification (P=0.006), invasion (P<0.001), focality (P=0.019) and the total number of CLN dissected (P<0.001) were all associated with CLNM in PTC patients. Compared with the non-CLNM group, the CLNM group had a higher proportion of male (29.4%), isthmus (20.6%), calcification presence (63.5%), Capsule +ETE (38.2%), multifocal (34.7%) and maximum tumor diameter≥20mm (18.2%), all of which had a positive effect on CLNM. On the contrary, age≥55 years old and microcarcinoma presence were both negatively related with CLNM (**Table 3**).

**Table 3:** Univariate analysis of the clinicopathological factors associated with CLN in PTC patients.

Group	without CLNM (n=245)	with CLNM (n=170)	t/U/χ^2^ value	P
Age	48.10 ± 11.312	41.11 ± 12.011	13841	0.000
<55	176 (71.8%)	143 (84.1%)	7.836	0.005
≥55	69 (28.2%)	27 (15.9%)		
Gender				
Male	46 (18.8%)	50 (29.4%)	5.801	0.016
Female	199 (81.2%)	120 (70.6%)		
Position				
Left	107 (43.7%)	54 (31.8%)	8.762	0.023
Right	106 (43.3%)	79 (46.5%)		
Bilateral	31 (0.4%)	2 (1.2%)		
Isthmus	1 (12.7%)	35 (20.6%)		
Maximum tumor diameter (mm)	9.13 ± 7.088	11.94 ± 7.530	15244.	0.318
<20	224 (91.4%)	139 (81.8%)	7.693	0.006
≥20	21 (8.6%)	31 (18.2%)		
Microcarcinoma				
No	65 (26.5%)	83 (48.8%)	20.777	0.000
Yes	180 (73.5%)	87 (51.2%)		
Calcification				
No	124 (50.6%)	62 (36.5%)	7.554	0.006
Yes	121 (49.4%)	108 (63.5%)		
A/T >1				
No	127 (51.8%)	85 (50.0%)	0.072	0.789
Yes	118 (48.2%)	85 (50.0%)		
Invasion				
No	198 (80.8%)	105 (61.8%)	17.533	0.000
Capsule + ETE	47 (19.2%)	65 (38.2%)		
Focality				
Unifocality	187 (76.3%)	111 (65.3%)	5.501	0.019
Multifocality	58 (23.7%)	59 (34.7%)		
The total number of CLN dissected	4.39 ± 4.566	6.40 ± 4.597	14329	0.000
FT3 (pmol/L)	4.29 ± 1.08	4.32 ± 0.69	19594.5	0.306
FT4 (pmol/L)	12.76 ± 2.02	12.67 ± 2.21	20754.5	0.953
TT3 (nmol/L)	2.80 ± 21.15	1.56 ± 1.57	20451.5	0.756
TT4 (nmol/L)	94.40 ± 23.98	93.58 ± 20.63	20187.5	0.596
TSH (mIU/L)	2.33 ± 2.91	2.25 ± 1.60	20138.5	0.568
TPOAb (IU/ml)	71.52 ± 193.88	52.151 ± 139.44	20333	0.682
TgAb (IU/ml)	61.07 ± 170.79	77.94 ± 195.45	20773	0.965
HT				
No	167 (68.2%)	131 (77.1%)	3.496	0.062
Yes	78 (31.8%)	39 (22.9%)		

### Multivariate analysis predictive risk factors of CLNM in PTC patients

Our multivariate Logistic analysis revealed that position(Right, OR=1.920, 95%CI 1.166-3.164; Bilateral, OR=9.393, 95%CI 0.607-145.408; Isthmus, OR=1.066, 95%CI 0.451-2.522; P=0.033), Capsule + ETE (OR=2.246, 95%CI 1.363-3.700, P=0.001), multifocal (OR=1.999, 95%CI 1.029-3.885, P=0.041) and the total number of CLN dissected (OR=1.143, 95%CI 1.083-1.206, P<0.001) were defined as independent risk factors for CLNM. Conversely, age≥55years old (OR=0.358, 95%CI 0.201-0.637, P<0.001), female (OR=0.433, 95%CI 0.257-0.730, P=0.002), microcarcinoma (OR=0.329, 95%CI 0.205-0.528, P<0.001) and HT (OR=0.412, 95%CI 0.234-0.727, P=0.002) were protective factors for CLNM (**Table 4**).

**Table 4:** Multivariate analysis of the clinicopathological factors associated with CLNM in PTC patients.

Variables	Subgroup	OR	95% CI	P
Age	<55	1		0.000
	≥55	0.358	0.201-0.637	
Gender	Male	1		0.002
	Female	0.433	0.257-0.730	
Position	Left	1		0.033
	Right	1.920	1.166-3.164	
	Bilateral	9.393	0.607-145.408	
	Isthmus	1.066	0.451-2.522	
Microcarcinoma	No	1		0.000
	Yes	0.329	0.205-0.528	
Invasion	No	1		0.001
	Capsule + ETE	2.246	1.363-3.700	
Number of lesions	Unifocal	1		0.041
	Multifocal	1.999	1.029-3.885	
The total number of CLN dissected		1.143	1.083-1.206	0.000
HT	No	1		0.002
	Yes	0.412	0.234-0.727	

**Figure 1:**
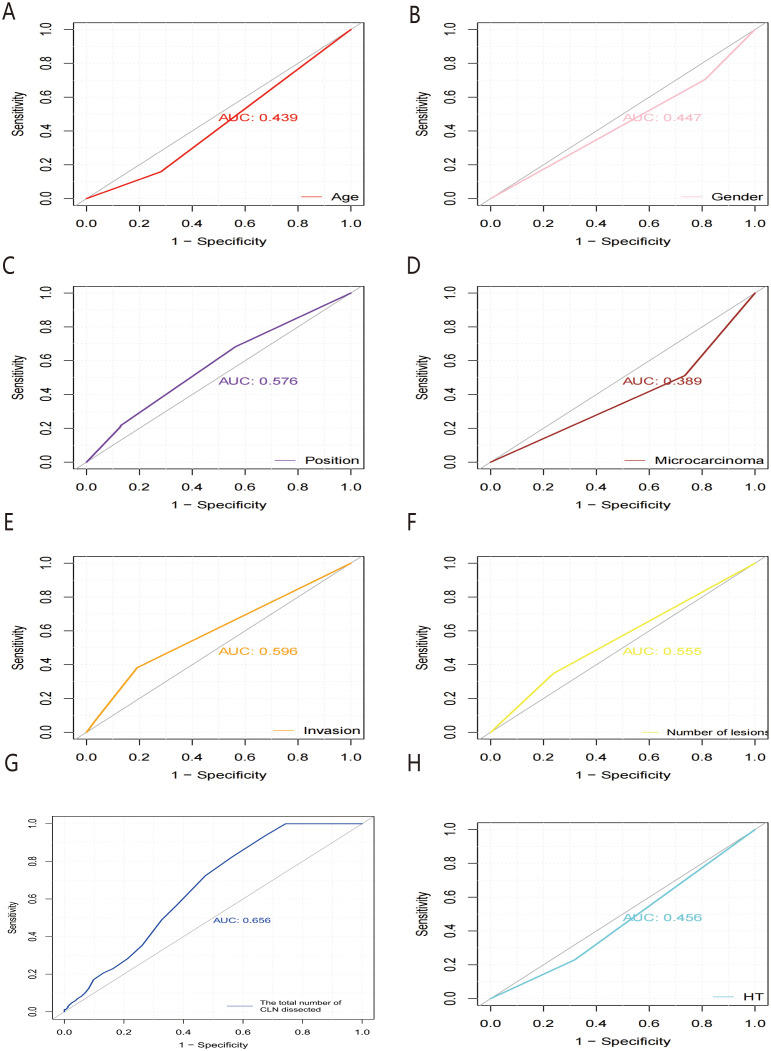
ROC curves of predicting the risk of CLNM in PTC patients by HT **(A)**, microcarcinoma **(B)**, the total number of CLN dissected **(C)**, position **(D)**, number of lesions **(E)**, gender **(F)**, invasion **(G)** and age stratification **(H)**, respectively. ROC, receiver operating characteristic; AUC, area under the ROC curve.

**Figure 2:**
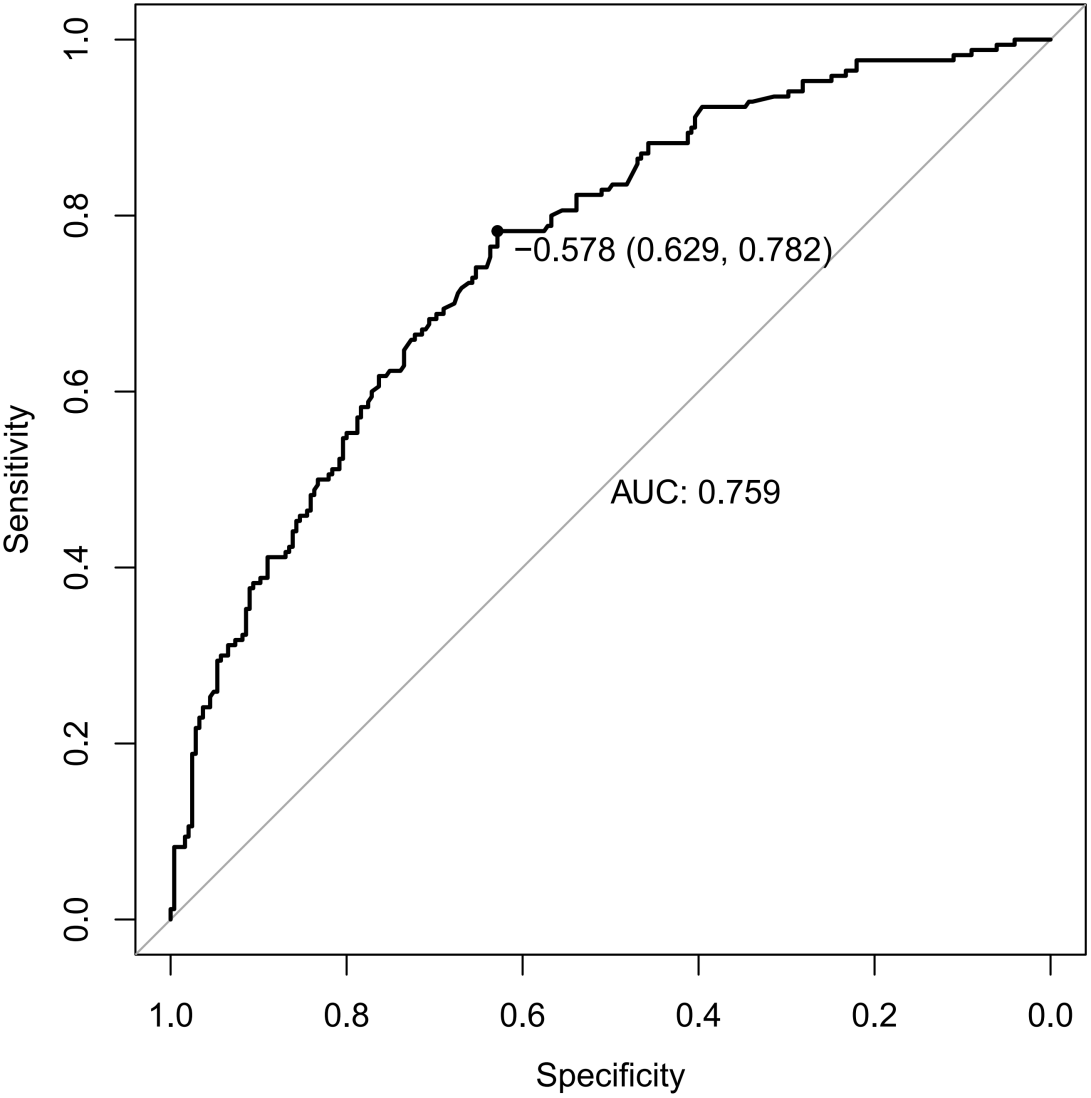
ROC curves of predicting the risk of CLNM in PTC patients by the model. ROC, receiver operating characteristic; AUC, area under the ROC curve.

**Figure 3:**
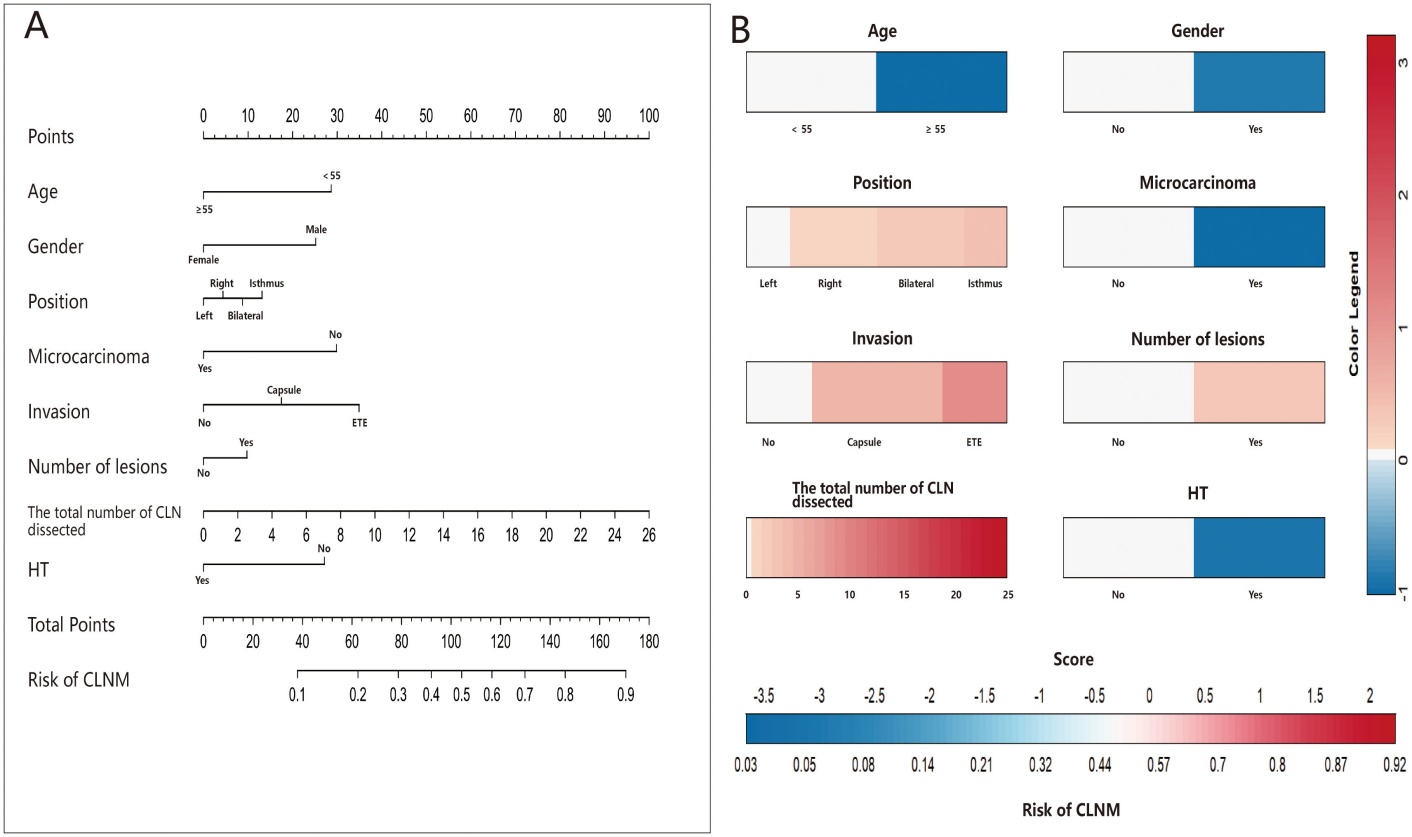
Nomograms **(A, B)** of the prediction model for the risk of CLNM in PTC patients.

**Figure 4:**
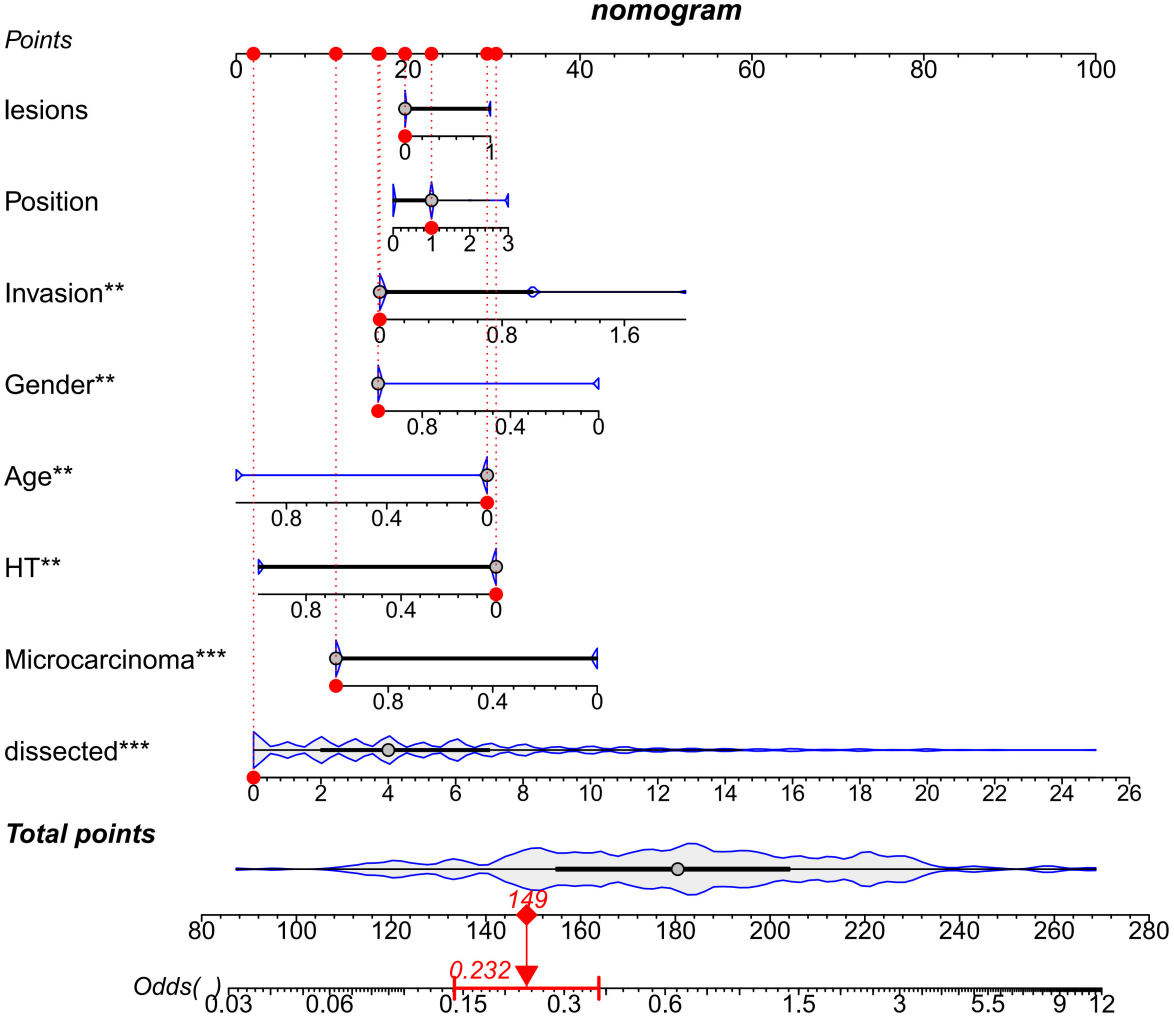
Dynamic Nomogram of the prediction model for the risk of CLNM in PTC patients.

**Figure 5:**
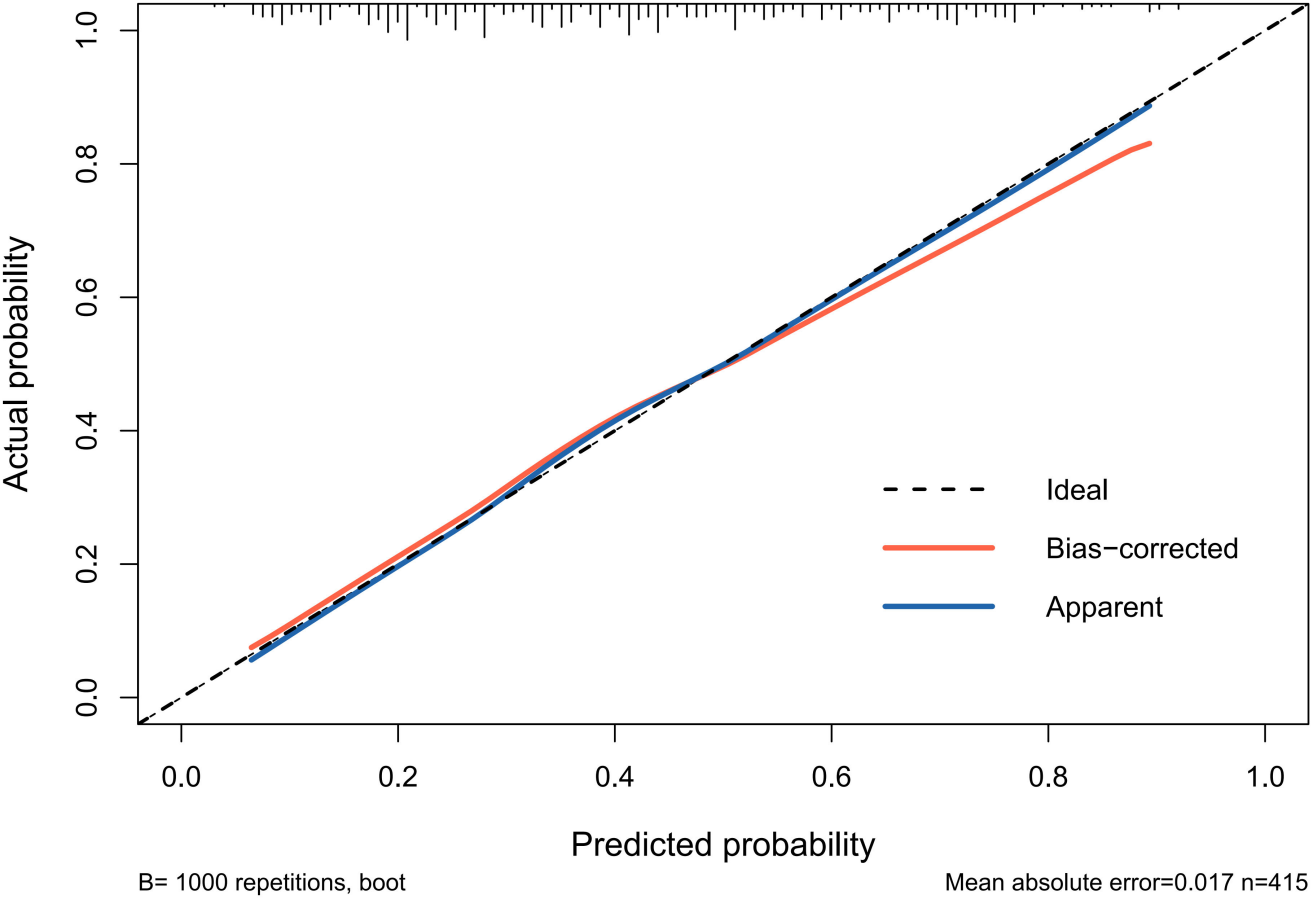
Calibration curve of the prediction model for the risk of CLNM in PTC patients.

**Figure 6:**
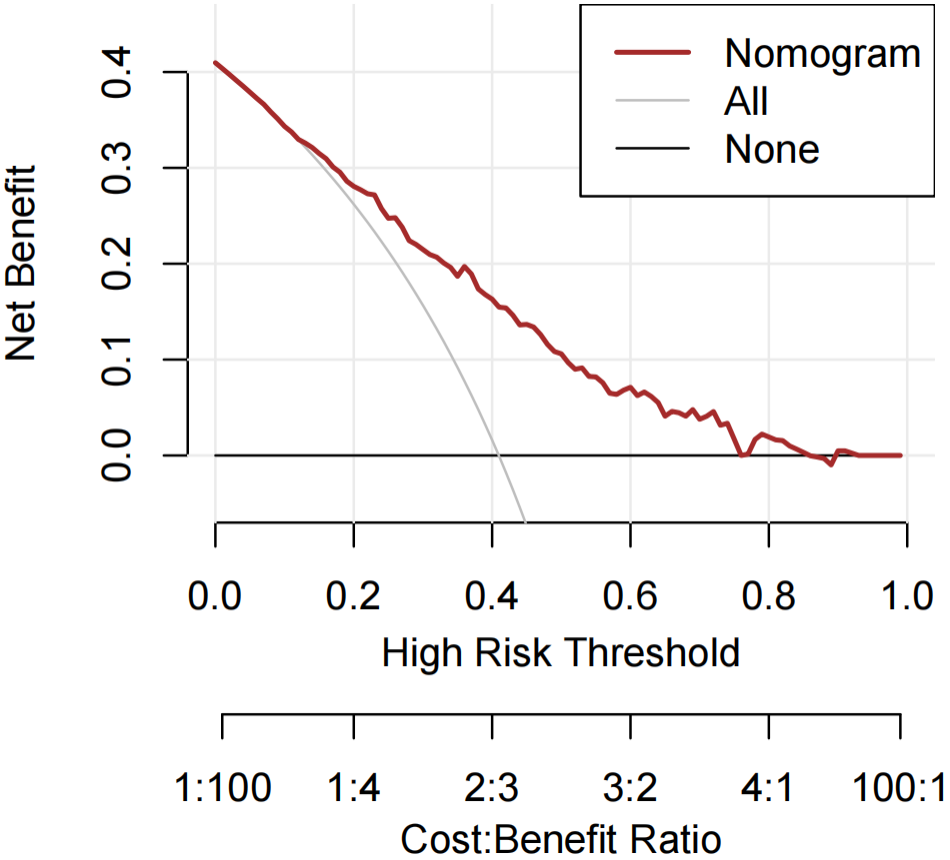
Decision curve analysis of the prediction model for the risk of CLNM in PTC patients.

Receiver operating characteristic (ROC) curves were drawn to predict the risk of CLNM in PTC patients by using the significant factors respectively (**Figure 1**). The respective values for the area under the curve (AUC) of age stratification, gender, position, microcarcinoma, invasion, number of lesions, the total number of CLN dissected and HT were respectively 0.439, 0.447, 0.576, 0.389, 0.595, 0.555, 0.656 and 0.456 (all P<0.1). On this basis, we use these 8 factors to build a prediction model and plot its ROC curve. The AUC of this model was 0.759, which was higher than the previous individual factors (**Figure 2**). Therefore, it demonstrates that the prediction model has a higher diagnostic value. To visualize the model, different versions of the nomogram were plotted to better illustrate the problem (**Figures 3**, **4**). The calibration curve revealed good predictive accuracy between the actual probability and predicted probability (**Figure 5**). To determine the net benefit of the nomogram, we built a decision curve analysis (DCA) (**Figure 6**). The curve indicated that the nomogram could be useful when the threshold probability was between 0.15 and 0.75. In the prediction model, the DCA’s net benefit was substantially higher.

### Multivariate linear regression analysis of risk factors of CLN positive rate in PTC patients

Based on multiple linear regression analysis, a new regression equation was established for predicting the risk of CLN positive rate in patients with PTC, which had statistical significance (F=5.750, P<0.001). Among them, age (β=-0.229, P<0.001), female gender (β=- 0.161, P=0.001), and microcarcinoma (β=- 0.244, P=0.001) exhibited negatively predictive effects; while invasion (β= 0.093, p=0.048) showed a positively predictive effect. Together, these variables collectively accounted for 16.3% of the variation in the CLN positive rate (**Table 5**).

**Table 5:** Multivariate Linear Regression Analysis about Risk Factors of CLN Positive Rate in PTC Patients.

Variables	B	β	t	95% CI	P	F	Adjusted R^2^
Age	-0.006	-0.229	-4.953	-0.009~-0.004	0.000	5.750	0.163
Gender	-0.128	-0.161	-3.448	-0.201~-0.055	0.001		
Position	0.006	0.017	0.298	-0.031~0.043	0.766		
Maximum tumor diameter (mm)	0.001	0.023	0.312	-0.005~0.007	0.755		
Microcarcinoma	-0.170	-0.244	-3.374	-0.269~-0.071	0.001		
Calcification	0.014	0.022	0.459	-0.048~0.076	0.647		
A/T>1	-0.022	-0.032	-0.671	-0.085~0.042	0.503		
Invasion	0.070	0.093	1.981	0.001~0.140	0.048		
Number of lesions	0.051	0.069	1.190	-0.033~0.135	0.235		
FT3	0.006	0.016	0.302	-0.032~0.044	0.763		
FT4	-0.011	-0.070	-0.930	-0.035~0.013	0.353		
TT3	-0.001	-0.044	-0.941	-0.003~0.001	0.347		
TT4	4.073E-5	0.003	0.038	-0.002~0.002	0.970		
TSH	-0.003	-0.022	-0.399	-0.018~0.012	0.690		
TPOAb	-9.824E-5	-0.051	-0.997	0.000~0.000	0.319		
TgAb	-8.258E-6	-0.004	-0.089	0.000~0.000	0.929		
HT	-0.068	-0.091	-1.694	-0.146~0.011	0.091		

## Discussion

Thyroid carcinoma is the most prevalent endocrine malignancy with soaring morbidity worldwide. As of 2018, it ranked ninth among all cancers globally (21). There are four common pathological types of thyroid carcinoma, with PTC being the most frequent type, accounting for approximately 80% of cases and generally having a favorable overall survival rate (22, 23). Nonetheless, Lim et al. (24) discovered that both morbidity and mortality of PTC have been gradually ramping up in recent years. The underlying reasons of this trend remain unclear. Currently, the academic community is actively investigating potential risk factors in hopes of addressing this issue.

HT, as a complex autoimmune thyroid disease, is characterized by diffuse lymphocytic infiltration (especially T cells) and follicular destruction leading to progressive atrophy and fibrosis of thyroid tissue, even to progressive damage, which is clinically manifested as obvious hypothyroidism (25). Some studies believe that its pathogenesis may be the combination of genetic susceptibility and environmental factors, resulting in the loss of immune tolerance, and subsequent autoimmune attack on thyroid tissue, ultimately the disease appears (26–28).

The relationship between PTC and HT has been the focus of extensive research. Investigations have sought to determine whether special clinicopathological features or prognostic implications arise when they coexist. Since the association between HT and PTC was first described in 1955 (14), numerous studies have confirmed that HT elevates the risk of developing PTC (29, 30). As Danis et al. (31)found, among 469 patients who underwent thyroidectomy, 54.9% were diagnosed with both PTC and HT, 33.1% with HT alone, and the remaining 12% with PTC alone (P<0.001).

This paper aims to analyze the clinical and pathological characteristics of PTC patients combined with HT through a retrospective study, and on this basis, further delves into the risk factors affecting central lymph node metastasis in PTC patients. We have obtained some novel insights and perspectives.

Firstly, this study found that the PTC+HT group had a higher proportion of female patients than the PTC group (P<0.05), indicating that female PTC patients are more likely to have coexisting HT. Similar results were gained by Heo et al. (32) through a retrospective analysis of case data. Cappellacci et al. (33) also believed that PTC patients with concurrent HT had a younger age of onset (P=0.4131) and a greater female predominance compared to those without HT (P<0.0001). In addition, our study revealed the proportion of A/T > 1 in the PTC+HT group (59.8%) was higher than that in the PTC group (44.6%), and there was a statistical difference between them (P=0.007). This result suggests that patients with concurrent HT are more prone to experience longitudinal growth of nodules, thereby increasing the risk of PTC.

Secondly, our investigation revealed that the mean values of preoperative TSH, TPOAb, and TgAb in the PTC+HT group were higher than those in the PTC group, and the latter two were more significant, with statistical significance (all P<0.001). Multivariate Logistic regression analysis further provided additional confirmation that HT was positively correlated with TSH, TPOAb, and TgAb. These results told us that the coexistence of HT can cause blood TSH, TPOAb, and TgAb to rise in PCT patients. Currently, it is an indisputable fact that the occurrence of thyroid cancer is related to elevated TSH levels in the blood. TSH, as a growth factor, can regulate the proliferation and function of thyroid cells under normal circumstances. When TSH exceeds the upper limit of the normal range, it is positively correlated with the malignant transformation rate of nodular goiter. Many studies have verified this view (34, 35).

However, the presence of HT in PTC patients could trigger the body’s autoimmune response mechanism, leading to the production of autoantibodies that specifically target thyroid antigens by immune cells. These autoantibodies mainly act on Thyroid peroxidase (TPO) and Thyroglobulin (Tg), resulting in atrophy and destruction of thyroid cells, even causing hypothyroidism. At this time, there is a decline in the production of thyroid hormones, which motivates the body to stimulate the pituitary gland to accelerate the secretion of TSH through the feedback regulation mechanism of the endocrine system, thus elevating the concentration of serum TSH (36, 37). Therefore, coexisting HT can promote the release of TSH, TPOAb, and TgAb in PCT patients, engendering an increase of their respective components in serum. Through research, Sakiz et al. (38) also observed that the TSH concentration in PTC patients with coexisting HT was significantly higher than that in patients without HT (1.71mIU/L vs 1.28 mIU/L, P=0.001).

Once again, through univariate analysis, the result found that the total number of CLN dissected in the PTC+HT group was higher than that in the PTC group, but the positive rate of CLN in the PTC+HT group was lower than that in the PTC group (P=0.000, P=0.007). Meanwhile, multivariate logistic regression analysis was used to validate that HT was positively associated with the total number of CLN dissected but negatively correlated with invasion. These findings show that PTC patients with HT are more prone to experience lymph node enlargement, yet ultimately the malignancy rate of the central lymph node is lower, which also reduces the invasiveness of PTC. Therefore, it is believed that HT not only increases the risk of developing PTC but also prevents further deterioration of PTC disease.

Hence, the growing evidence also illustrates that the chronic inflammatory process of HT confers a protective effect on PTC progression. Likewise, a retrospective cohort study from a large medical center in China confirmed that papillary thyroid microcarcinoma (PTMC) patients with coexisting HT have a significantly lower incidence of lymph node metastasis in the central neck (40.9% vs 56.2%, P<0.001) and lateral neck (11.6% vs 14.2%, P=0.016), which indicated that patients with HT had fewer aggressive features and better prognosis (39). However, there are some studies with controversial conclusions. For example, Zeng et al. (18) uncovered that among PTC patients, those with coexisting HT have a higher incidence of central lymph node metastasis compared to non-HT patients (39.2% vs 31.4%, P=0.043). A retrospective cohort study also showed HT was an independent risk factor in predicting CLNM in PTC patients (40).

Thus far, only a handful of studies have investigated the impact of HT on CLNM in PTC. In our study, we compared the data between the non-CLNM group and the CLNM group using univariate analysis, then verified that age, gender, position, microcarcinoma, calcification, invasion, focality, and the total number of CLN dissected were all associated with CLNM in PTC patients, all of which were statistically significant. Additionally, we also acquired a captivating outcome. Although the data showed that the PCT patients with HT in the CLNM group were significantly less than those in the non-CLNM group, indicating that the combination of HT might decrease the occurrence of CLNM in PCT patients, unfortunately, this difference did not reach statistical significance. Our data showed the PCT patients with HT in the CLNM group were significantly fewer than those in the non-CLNM group, indicating that the combination of HT may reduce the occurrence of CLNM in PCT patients. Unfortunately, this difference did not reach statistical significance.

Furthermore, we conducted a multivariate logistic regression analysis, which manifested that position, Capsule + ETE, multifocality, and the total number of CLN dissected were independent risk factors for CLNM. Conversely, age ≥ 55 years old, female, microcarcinoma, and HT were identified as protective factors against CLNM. These results are consistent with the previous comparison between the HT Group and PTC+HT Group, providing additional evidence that HT is more susceptible to causing lymph node enlargement in PTC, but not true lymph node metastasis. Through a study of 444 PTC patients, Wang et al. (41) concluded that the autoimmune response of HT seems to reduce the occurrence of CLNM in PTC patients. Meanwhile, age<55 years and tumor size ≥10mm are identified as independent risk factors for CLNM. Building upon these results, Battistella et al. (42) further discovered that the PTC-HT group had smaller tumor size, lower invasiveness, and fewer lymph node metastasis, simultaneously with a higher rate of early tumor diagnosis. Moreover, we have also identified the risk factors that are prone to induce CLNM in PTC: Isthmus, Capsule + ETE, multifocality, age<55 years old, and male gender. Some studies have also yielded similar results. For instance, Liu et al. (43) conducted a clinical data analysis of 966 patients and verified that male, age<45 years old, tumor size>1.0cm, ETE, and microcalcification were independent risk factors for CLNM. Even, some researchers put these high-risk factors together to establish a model that might serve as a more comprehensive theoretical basis for clinical assessment of the risk of CLNM (44). We also constructed a similar prediction model based on the above independent factors to judge the probability of CLNM occurrence. The results showed that the model has a high diagnostic value and certain generalization significance.

Finally, we performed a multiple linear regression analysis, incorporating all potential factors related to the CLN positive rate. The results stated that age, female, and microcarcinoma exhibited negative predictive effects, whereas invasion showed a positive predictive effect. Combining the previous logistic regression analysis results on the risk factors of CLNM, we believe that age<55 years old, male, position, Capsule+ETE, and multifocality are all potential risk factors for CLNM. However, among these factors, the presence of Capsule + ETE is the most influential one that can ultimately affect the rate of CLNM transfer. Therefore, the stronger the invasiveness of carcinoma in PTC patients, the higher the likelihood of CLNM occurrence, eventually leading to a higher ratio of CLNM.

## Conclusion

In summary, our experimental conclusion is that female PTC patients are more prone to concurrent HT, which elevates the levels of TSH, TPOAb, and TgAb in the blood of PTC patients. HT not only promotes the longitudinal growth of nodules and PTC development, but also reduces the risk of invasion and CLNM. Therefore, we posit that the impact of HT on PTC patients is a “double-edged sword”. Isthmus, Capsule + ETE, multifocality, age < 55 years old, and male are high-risk factors for CLNM in PTC, while HT is regarded as a protective factor. Capsule + ETE is the primary risk factor affecting the CLN positive rate. Our results are consistent with the results of most similar studies, and our data confirm that HT reduces the risk of CLNM. According to our conclusions, we suggest that patients with clinically diagnosed HT should actively monitor and manage the levels of TSH, TPOAb, and TgAb, reducing or avoiding the conversion to PTC. Additionally, if the aforementioned high-risk factors are absent in preoperative ultrasound and clinical data analysis, there is no need for extensive prophylactic central lymph node dissection during surgery when HT is combined with PTC. We hope that the conclusions of this study will offer valuable insights and guidance to clinicians.

## Limitations

Nevertheless, in light of this study primarily involving single-center data analysis, there may be certain limitations that could lead to biases in the final research conclusions. In the future, we will continue to make efforts to carry out some studies on large sample sizes, multiple centers, and molecular genetics, leveraging the experiences and achievements of previous researchers, to gain a deeper understanding about the pathological transformation mechanisms of how HT affects the patients of PTC and bring more benefits to thyroid disease patients.

## Data Availability

The original contributions presented in the study are included in the article/**Supplementary Material**. Further inquiries can be directed to the corresponding author.
